# Enhanced Stability of *Lactobacillus paracasei* Aspartate Ammonia-Lyase via Electrospinning for Enzyme Immobilization

**DOI:** 10.3390/polym17030270

**Published:** 2025-01-22

**Authors:** Chun-Yen Hsieh, Yi-Hao Huang, Yu-Ting Yu, Kai-Wei Chang, Yung-Ju Chen, Lu-Sheng Hsieh

**Affiliations:** 1 Department of Pathology and Laboratory Medicine, Shin Kong Wu Ho-Su Memorial Hospital, Taipei City 111, Taiwan; t012874@ms.skh.org.tw; 2 Department of Food Science, College of Agriculture and Health, Tunghai University, Taichung 407, Taiwan; yihaoh06@gmail.com (Y.-H.H.); tiffycat920@gmail.com (Y.-T.Y.); kaiwei1005@icloud.com (K.-W.C.)

**Keywords:** aspartate ammonia-lyase, electrospinning, immobilized enzyme, *Lactobacillus paracasei*, nanofiber membranes

## Abstract

This study investigates the immobilization of *Lactobacillus paracasei* AAL (LpAAL) protein onto polyvinyl alcohol/nylon 6/chitosan nanofiber membranes using dextran polyaldehyde as a biodegradable cross-linker. Immobilization enhanced the enzyme’s stability, shifting its optimal reaction conditions from 40 °C to 45 °C and pH from 8.0 to 8.5. While immobilization slightly reduced its catalytic efficiency, it significantly improved enzyme stability and reusability. The immobilized enzyme retained 85% of its initial activity after 7 days of storage at room temperature, compared to 55% for the free enzyme. Reusability tests demonstrated that immobilized LpAAL protein maintained approximately 50% of its activity after six consecutive reaction cycles, highlighting its robustness over repeated use. These results underscore the advantages of nanofiber-based immobilization in enhancing enzyme stability and utility for industrial applications, offering a practical approach to overcoming the limitations associated with free enzyme systems.

## 1. Introduction

Aspartate ammonia-lyase (AAL, EC 4.3.1.1) is an enzyme extensively studied in industrial biotechnology, due to its ability to catalyze the non-oxidative deamination of L-aspartate to fumarate [1,2]. Found across a wide range of microorganisms, AAL plays a critical role in nitrogen and organic acid metabolism [3,4,5]. Belonging to the lyase family, AAL exhibits remarkable substrate specificity. Initially thought to exclusively catalyze L-aspartate [6,7], subsequent studies have shown that certain microbial AALs possess a broader substrate range. For instance, AAL from *Pseudomonas fluorescens* can catalyze the conversion of L-phenylalanine to *trans*-cinnamic acid [8]. Similarly, *Lactobacillus paracasei* AAL demonstrates the ability to catalyze both L-tyrosine to *p*-coumaric acid and L-phenylalanine to *trans*-cinnamic acid, underscoring its substrate versatility [9]. These catalytic properties, which vary significantly across microbial sources, highlight AAL’s broad potential for diverse applications.

Structural and mechanistic studies have shed light on key aspects of AAL functionality. Enzymatic activity depends on the presence of Mg^2+^ and alkaline conditions, with a serine residue playing a pivotal role in the catalytic mechanism [1,10]. AAL from *Bacillus* sp. (AspB) demonstrates significant thermal stability and independence from Mg^2+^ for activity, highlighting its adaptability under diverse environmental and industrial conditions [11,12]. Such characteristics make AAL a promising enzyme for industrial applications, particularly in the synthesis of L-aspartate, which serves as a precursor for artificial sweeteners, aspartame, food additives, and in pharmaceutical manufacturing [12,13]. Advances in protein engineering, including site-directed mutagenesis and computer-aided design, have further enhanced AAL’s substrate specificity and catalytic efficiency, enabling its application in synthesizing enantioselective amino acids from non-natural substrates [14]. These developments underscore AAL’s utility as a vital tool in enzyme engineering and metabolic pathway optimization for sustainable industrial processes [5,15].

Immobilization techniques are crucial for addressing challenges associated with enzyme stability, reusability, and catalytic efficiency in industrial applications [16]. Early efforts focused on immobilizing whole cells, expressing AAL onto carriers such as polyacrylamide, polyurethane, or carrageenan. These approaches enhanced enzyme stability, productivity, and cost-effectiveness, but faced limitations due to restricted enzyme accessibility and substrate diffusion [17,18,19]. To overcome these constraints, later methods employed purified AAL immobilized on carriers like epoxy resins, amino resins, polyacrylamide gels, and magnetic nanoparticles, leading to significant improvements in catalytic performance and operational stability [1,2,20].

Recent advancements in immobilization include system optimization strategies, such as overexpressing AAL in the psychrophilic bacterium *Shewanella livingstonensis* combined with heat treatment to remove interfering enzymes, achieving a conversion rate of 99.3% [21]. Furthermore, co-immobilization of AAL with transaminases on ReliZyme^®^ carriers has demonstrated its industrial potential for producing L-phenylalanine, an important intermediate in pharmaceutical and food applications [22]. These innovations illustrate the versatility and scalability of AAL immobilization techniques for industrial needs.

Nanotechnology has revolutionized enzyme immobilization by introducing nanofibers as advanced carriers that address limitations in enzyme stability and performance [23]. Nanofibers, characterized by their high surface area-to-volume ratio, abundant pore structures, customizable surface functionalities, and excellent mechanical properties, offer unparalleled advantages for enzyme immobilization. These attributes enhance enzyme activity, tolerance to extreme conditions, and storage stability, making nanofibers ideal for biocatalysis [24,25,26].

**Figure 1:**
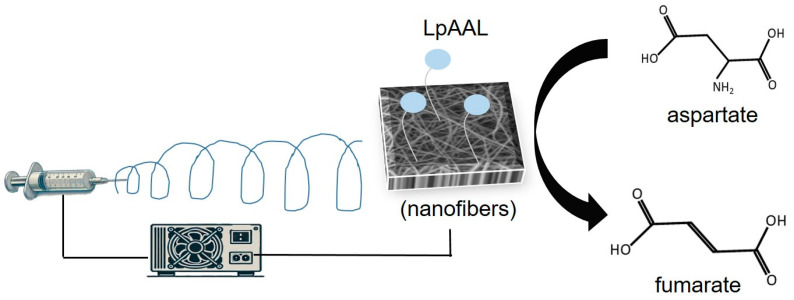
Schematic representation of LpAAL immobilization and catalytic reaction. The schematic represents the immobilization of LpAAL protein on nanofiber membranes and the catalytic conversion of L-aspartic acid (Asp) to fumarate, facilitated by the immobilized enzyme.

Electrospinning, a widely adopted method for nanofiber synthesis, employs high electric fields to produce continuous polymer fibers. This cost-effective and efficient technique generates composite fibers with large surface areas, facilitating improved mass transfer between substrates and enzyme-active sites [27,28]. Additionally, electrospun nanofibers exhibit excellent reusability and easy recoverability, further enhancing their value as biocatalyst carriers [29,30]. Studies have consistently demonstrated that nanofiber carriers improve the activity and stability of immobilized enzymes, addressing industrial demands for efficiency, reliability, and cost-effectiveness [31,32]. These advancements position nanofibers as a promising platform for next-generation industrial biocatalysis. This study aims to explore the application potential of polyvinyl alcohol (PVA), nylon 6, and chitosan nanofiber membranes for immobilizing *Lactobacillus paracasei* AAL. Previous studies have demonstrated that these materials significantly enhance the stability and reusability of immobilized enzymes, including phenylalanine ammonia-lyase and glutamate decarboxylases, by leveraging their high surface area-to-volume ratio and customizable functionalities [30,33,34]. These attributes are hypothesized to similarly enhance the performance of immobilized LpAAL. The unique substrate specificity and catalytic efficiency of *L. paracasei* AAL make it an excellent candidate for industrial applications [9]. This research seeks to optimize the stability, catalytic efficiency, and industrial applicability of immobilized AAL through nanofiber membrane technology (Figure 1), contributing to the advancement of robust and sustainable enzyme immobilization techniques.

## 2. Materials and Methods

### 2.1. Chemical Reagents

The reagents for L-aspartic acid, protein electrophoresis, and molecular biology operations have been described in previous publications [9,35,36]. The materials used for electrospinning have also been reported in earlier studies [30,33,34].

### 2.2. Expression of Recombinant LpAAL in Escherichia coli BL21

Recombinant *Escherichia coli* BL21 containing the pMAL-c2x-LpAAL plasmid was cultivated in 250 mL of LB medium (composed of 0.5% yeast extract, 1% tryptone, and 1% NaCl) enriched with 100 μg/mL ampicillin at a temperature of 30 °C, while shaking at 200 rpm overnight [6]. Protein expression was initiated by the addition of isopropyl b-D-1-thiogalactopyranoside (IPTG) to a final concentration of 0.1 mM, once the optical density at 600 nm (OD_600_) reached 0.6–1.0. Subsequently, the cells were incubated at 30 °C with vigorous shaking for an additional duration of 4 h [9].

### 2.3. Preparations of LpAAL Enzymes

The *E. coli* cell pellet was re-suspended in 20 mL of 1× lysis buffer (comprising 50 mM Tris-HCl, pH 7.5, 10 mM imidazole, 100 mM NaCl, and 1 mM PMSF) and subsequently subjected to disruption via sonication, employing a Branson cell disruptor. The resultant cell lysates containing LpAAL protein were then introduced into an amylose affinity chromatography column, from which they were ultimately eluted using a buffer supplemented with 10 mM maltose.

### 2.4. SDS-Polyacrylamide Gel Electrophoresis

Protein samples were prepared by heating with SDS-containing loading buffer at 95 °C for 5 min. Samples were run on a 12% polyacrylamide gel in Tris-glycine buffer at 120 V. Following electrophoresis, the gel was stained with Coomassie Brilliant Blue R-250 and destained with 10% methanol. Protein markers were used for molecular weight estimation (Bio-Rad, Hercules, CA, USA).

### 2.5. AAL Activity Assay

The enzymatic activity of AAL was evaluated through the quantification of fumaric acid production, with absorbance measured at 240 nm, utilizing fumaric acid as a reference standard [3,8]. The reaction mixture comprised 50 mM Tris-HCl (pH 8.0), 11 mM L-aspartic acid, and a suitable quantity of enzyme, achieving a total volume of 1.0 mL. The reaction was maintained at an incubation temperature of 40 °C for a duration of 5 min [9].

### 2.6. Electrospinning of PVA/Nylon 6/CS Nanofiber Membranes

Polymer solutions were formulated using polyvinyl alcohol (PVA) at a concentration of 4% (*w*/*v*), nylon 6 at 8% (*w*/*v*), and chitosan (CS) at 1% (*w*/*v*), which were dissolved in formic acid and subjected to agitation in a water bath maintained at 60 °C for 1 h with magnetic stirring. Upon cooling to room temperature, the solutions were subsequently transferred into a 5 mL syringe (21 G, internal diameter of 13 mm). This syringe was then connected to an NE-300 syringe pump (New Era Pump Systems, Farmingdale, NY, USA) to regulate a consistent flow rate of 360 mL/h. The spinneret needle was linked to a high-voltage power supply (Model FES-COS, Falco, New Taipei City, Taiwan) and charged to 13.8 kV. The electrospinning process was conducted with the spinneret’s tip positioned at 10 cm from the collector plate [33,34]. The resulting nanofiber membranes were gathered on a roller collector that was lined with baking paper.

### 2.7. Preparation of Dextran Polyaldehyde Cross-Linker and Immobilization of LpAAL Protein on Nanofiber Membranes

The synthesis of the dextran polyaldehyde cross-linker was conducted according to the established methodology delineated by Wu et al. [34]. In summary, 1 g of dextran was reacted with 2.3 g of sodium metaperiodate in 30 mL of a 50 mM sodium phosphate buffer (pH 6.0), under conditions devoid of light for a duration of 2 h. The oxidation reaction was subsequently halted through the addition of 100 mL of ethylene glycol, followed by an overnight dialysis in the same buffer, to eliminate any unreacted reagents. The resultant dextran polyaldehyde cross-linker was preserved at 4 °C for future applications. During the immobilization phase, 3 mg of nanofiber membrane underwent cross-linking with purified recombinant LpAAL protein, while being subjected to vigorous agitation at 150 rpm. The conditions for immobilization were refined by adjusting the concentration of the dextran polyaldehyde cross-linker (1–5%) and the incubation duration (3–24 h).

### 2.8. Enzyme Biochemical Properties and Kinetics

The enzymatic activity of free and immobilized LpAAL proteins was measured following the standard protocol outlined in Section 2.5. Optimal reaction conditions, including temperature and pH, were determined by performing activity assays at varied temperatures (30–50 °C) and pH levels (7.0–9.0), using appropriate buffer systems.

To analyze the kinetic parameters of free and immobilized LpAAL proteins, reaction mixtures were prepared with L-aspartic acid concentrations ranging from 0 to 20 mM. Substrate saturation curves were generated after 5 min of incubation at 40 °C. The Michaelis–Menten equation [37] and double reciprocal plot [38] were used to calculate the kinetic parameters, including *K*_m_ and *k*_cat_, for both forms of the enzyme.

### 2.9. Storage Stability Assay

The stability of both free and immobilized LpAAL proteins during storage was assessed by subjecting the samples to an ambient temperature for durations spanning from 0 to 7 days. The enzymatic activity was quantified under controlled reaction conditions (50 mM Tris-HCl buffer, pH 8.0, supplemented with 11 mM L-aspartic acid) following a 5 min incubation period, to evaluate the enzyme’s temporal stability. This method was adapted from a previous study [39], with modifications to the enzymatic activity assay to align with the requirements of this research.

### 2.10. Reusability of Immobilized LpAAL Protein

The reusability of immobilized LpAAL protein was assessed under standardized reaction conditions, using 50 mM Tris-HCl buffer (pH 8.0) with 11 mM L-aspartic acid as the substrate. After a 5 min incubation, the residual AAL activity was measured to evaluate the temporal stability of the enzymes for six consecutive cycles.

### 2.11. Statistical Analysis of Data

All experimental procedures were conducted autonomously in triplicate, with the outcomes expressed as the mean ± standard deviation (SD, denoted by error bars). The visualization of the data and subsequent statistical evaluation were performed utilizing one-way analysis of variance (ANOVA) within SigmaPlot software (version 11.0, Systat Software Inc., San Jose, CA, USA).

## 3. Results and Discussion

### 3.1. Construction of the LpAAL Expression System

**Figure 2:**
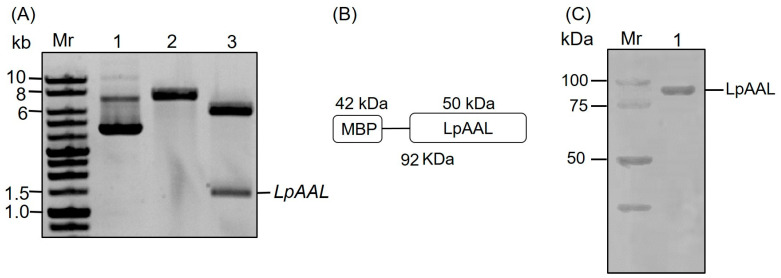
Preparation of recombinant LpAAL protein. (**A**) DNA gel electrophoresis of pMAL-c2x-LpAAL plasmid. Mr: 1 kb DNA marker. Lane 1: Undigested pMAL-c2x-LpAAL plasmid. Lane 2: Linearized pMAL-c2x-LpAAL plasmid with *Eco*R1 digestion. Lane 3: Double-digested pMAL-c2x-LpAAL plasmid with *Eco*R1 and *Bam*H1. (**B**) Schematic representation of the recombinant LpAAL protein conjugated with maltose binding protein (MBP). (**C**) SDS-PAGE analysis of recombinant LpAAL protein expressed in *E. coli*. Mr: Protein molecular weight marker. Lane 1: Purified recombinant LpAAL protein.

The agarose gel electrophoresis results demonstrated the successful construction and digestion of the pMAL-c2x-LpAAL plasmid. Lane 1 represents the undigested plasmid, exhibiting a distinct high-molecular-weight band corresponding to the circular plasmid (Figure 2A). Lane 2 displays the plasmid linearized with *Eco*R1, revealing a single band indicative of the full plasmid length (approximately 8040 bp). Lane 3 shows the plasmid digested with both *Eco*R1 and *Bam*H1, producing two fragments of approximately 6645 bp and 1395 bp, corresponding to the vector backbone and the *LpAAL* insert, respectively (Figure 2A). These findings suggest the successful integration of the *LpAAL* gene into the vector and its correct orientation between the restriction sites.

The recombinant LpAAL protein expressed using the pMAL-c2x-LpAAL system includes a 50 kDa LpAAL protein fused with a 42 kDa maltose binding protein (MBP) tag (Figure 2B). The MBP fusion protein facilitates purification and enhances protein solubility, as reported in prior studies [40,41]. On SDS-PAGE, the recombinant protein migrated between 75 and 100 kDa, aligning with the expected molecular weight of approximately 90 kDa (Figure 2C). Expression of untagged LpAAL recombinant protein was prone to form inclusion body [9]. The selection of the pMAL-c2x vector was informed by its proven ability to address solubility challenges in direct AAL expression without compromising enzymatic activity [9].

### 3.2. Optimized Cross-Linking Conditions for Immobilization of LpAAL Protein

**Figure 3:**
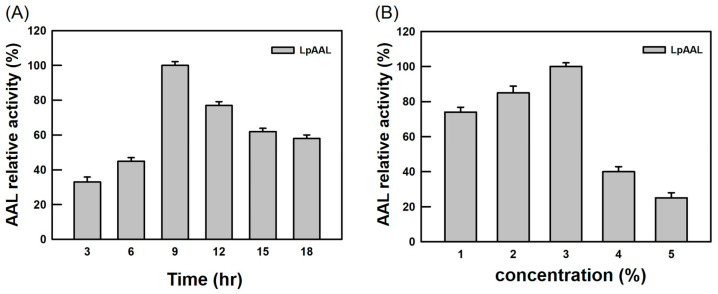
Optimization of cross-linking conditions for the immobilization of LpAAL protein on PVA/nylon 6/CS nanofiber membranes. The enzymatic activity of LpAAL protein was measured under varying cross-linking durations (**A**) and dextran polyaldehyde concentrations (**B**). Each experiment was conducted three times, and the results were presented as the mean ± standard deviation (S.D., depicted by error bars). The highest observed activity in each set was standardized to 100%.

Dextran polyaldehyde was used as a biodegradable cross-linker to immobilize enzymes on nanofiber membranes by forming covalent bonds with amino groups on the enzymes and nanofiber membranes [30,42,43]. The results indicated that the optimal enzymatic activity of LpAAL was achieved under cross-linking conditions of 9 h (Figure 3A) and 3% dextran polyaldehyde (Figure 3B). However, a decline in enzymatic activity was observed under excessive cross-linking conditions, suggesting that both the duration and concentration of the cross-linker significantly influence enzyme activity and stability. This reduction in activity may result from steric hindrance caused by over-cross-linked matrices and structural distortion of the enzyme, leading to a loss of catalytic function [42,44]. Such findings emphasize the importance of adjusting cross-linking parameters in enzyme immobilization to maintain functionality while ensuring stability, offering critical insights for the effective application of immobilized enzymes in industrial processes.

### 3.3. Determination of Optimal Reaction Conditions for Free and Immobilized LpAAL Proteins

**Figure 4:**
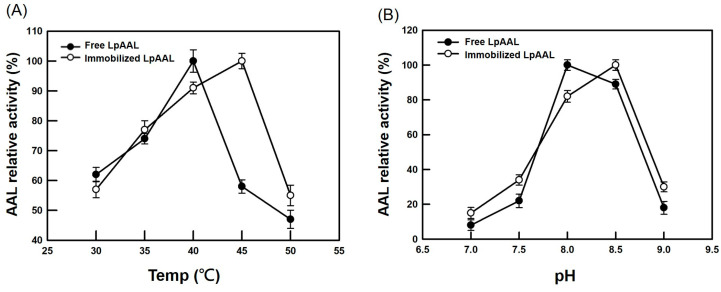
Optimal reaction conditions for free and immobilized LpAAL proteins. (**A**) The effect of temperature on enzymatic activity was evaluated across a range of 30–50 °C. (**B**) The effect of pH on enzymatic activity was assessed within a pH range of 7.0–9.0. Each experiment was conducted three times, and the results were presented as the mean ± standard deviation (S.D., depicted by error bars). The highest observed activity in each set was standardized to 100%.

The optimal reaction temperature and pH for free and immobilized LpAAL proteins were evaluated. Free LpAAL protein showed maximum activity at 40 °C, while immobilized LpAAL protein shifted to 45 °C (Figure 4A), suggesting enhanced thermal stability due to immobilization. Similarly, free LpAAL protein exhibited peak activity at pH 8.0, whereas immobilized LpAAL protein showed optimal activity at pH 8.5 (Figure 4B). These changes highlight the influence of immobilization on the enzyme’s microenvironment and substrate interactions, emphasizing its role in enhancing stability. By anchoring enzyme molecules within a porous solid matrix, immobilization provides structural support and shields them from deactivating factors such as extreme pH conditions and other destabilizing agents [45,46]. This structural stabilization explains the observed shifts in optimal temperature and pH, showcasing the benefits of immobilization in maintaining enzyme activity.

These results underscore the advantages of immobilized LpAAL protein for industrial applications. Enhanced thermal and pH stability broadens its usability across diverse reaction conditions, making it a more robust catalyst. This improved stability enables efficient performance under extreme conditions, increasing its practical value for industrial biocatalysis requiring high durability and prolonged enzymatic activity.

### 3.4. Kinetic Parameters of Free and Immobilized LpAAL Proteins

The kinetic analysis revealed differences in enzymatic efficiency between free and immobilized LpAAL proteins (Table 1). Free LpAAL protein exhibited a higher *k*_cat_ (13 s^−1^) compared to immobilized LpAAL protein (7.3 s^−1^), indicating a faster catalytic turnover for the free enzyme. However, immobilization led to an increased *K*_m_ value (6.3 mM) compared to free LpAAL (5.2 mM), suggesting a reduced substrate affinity post immobilization. The catalytic efficiency (*k*_cat_/*K*_m_) of immobilized LpAAL protein decreased to 1.2 s^−1^ mM^−1^, approximately half of that observed for free LpAAL protein (2.5 s^−1^ mM^−1^). These results reflect the structural and functional changes associated with enzyme immobilization. The reduction in *K*_m_ and *k*_cat_/*K*_m_ can be attributed to the cross-linking effect of restricted substrate permeability [30,47]. Additionally, immobilization may introduce microenvironmental changes or structural distortions to the enzyme, altering its intrinsic properties. Such changes can affect enzyme behavior and stability, occasionally leading to reduced catalytic performance [48]. These observations emphasize the importance of selecting the appropriate immobilization techniques and optimizing cross-linking conditions to minimize adverse effects, while ensuring the benefits of enhanced stability and reusability.

### 3.5. Storage Stability of Free and Immobilized LpAAL Proteins

**Figure 5:**
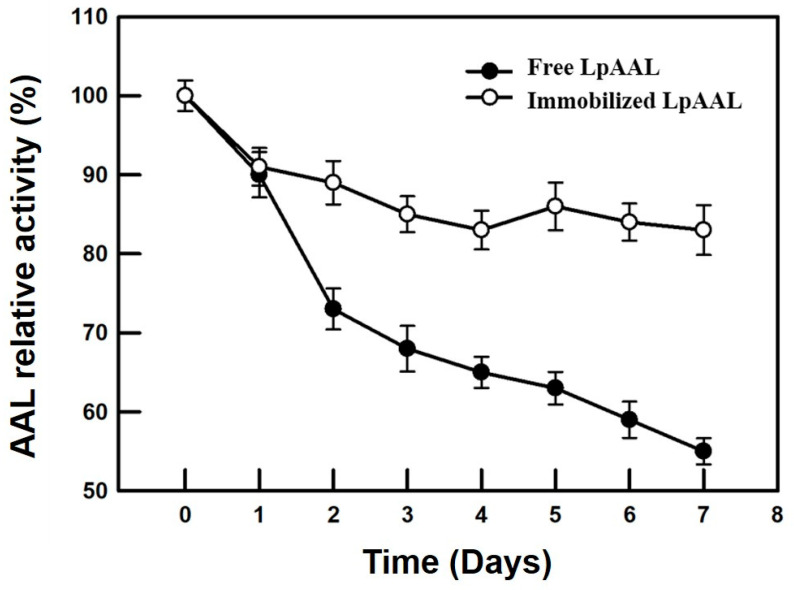
Storage stability of free and immobilized LpAAL proteins. The storage stability of free and immobilized LpAAL was assessed at room temperature over a 7-day period, with enzymatic activity expressed as a percentage of the initial activity. Each experiment was conducted three times, and the results were presented as the mean ± standard deviation (S.D., depicted by error bars).

The storage stability of free and immobilized LpAAL was assessed over 7 days at room temperature. The immobilized LpAAL protein retained significantly higher enzymatic activity compared to the free enzyme throughout the storage period (Figure 5). On day 7, the immobilized enzyme maintained approximately 85% of its initial activity, whereas the free enzyme showed a marked decline, retaining only around 55% of its original activity. This enhanced stability can be attributed to the immobilization process, which protects the enzyme from environmental deactivation factors and denaturation [42]. The improved storage stability of immobilized LpAAL protein highlights its potential for prolonged use in industrial applications. By stabilizing the enzyme structure through immobilization on a nanofiber membrane, it becomes less susceptible to activity loss over time.

### 3.6. Reusability of Immobilized LpAAL Protein

**Figure 6:**
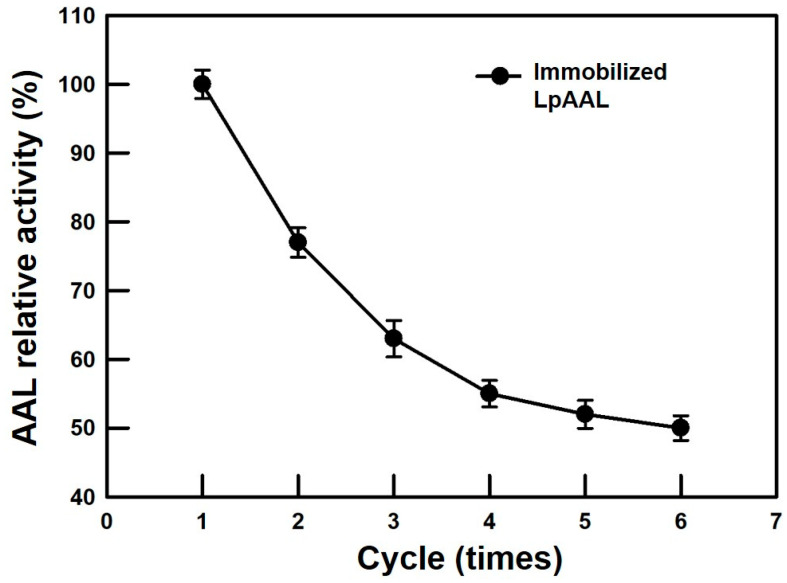
Reusability of immobilized LpAAL protein. The relative enzymatic activity of immobilized LpAAL was measured over six consecutive reaction cycles to evaluate its reusability, with enzymatic activity expressed as a percentage of the initial activity. Each experiment was conducted three times, and the results were presented as the mean ± standard deviation (S.D., depicted by error bars).

The reusability of immobilized LpAAL protein was evaluated over six reaction cycles, showing a gradual decline in relative enzymatic activity. After the first cycle, the enzyme retained approximately 80% of its activity, decreasing to around 60% by the third cycle and stabilizing at approximately 50% after six cycles (Figure 6). This observed activity loss could be attributed to partial enzyme leaching, structural destabilization, or conformational changes during repeated use [30,33]. Despite the decline, the immobilized enzyme demonstrated reasonable stability, underscoring its potential for repeated applications in industrial processes. This result highlights the advantage of immobilization in extending enzyme utility, while emphasizing the need for optimization to minimize activity loss over extensive reuse.

## 4. Conclusions

This study demonstrated that immobilizing LpAAL protein on PVA/nylon 6/chitosan nanofiber membranes enhances its stability and reusability. The immobilized enzyme retained significant activity under optimal conditions and prolonged storage, making it a robust candidate for industrial applications. Despite slight reductions in catalytic efficiency, the advantages of immobilization, including improved structural stability and extended usability, support its potential as an effective strategy for industrial biocatalysis. However, the observed decrease in enzyme activity highlights the importance of optimizing immobilization conditions and exploring alternative cross-linking agents and electrospinning parameters to enhance enzyme performance.

## Figures and Tables

**Table 1:** Kinetic parameters of free and immobilized LpAAL proteins. The table presents the kinetic parameters *k*_cat_, *K*_m_, and *k*_cat_/*K*_m_ for both free and immobilized LpAAL proteins, with references provided for comparison.

Enzyme	*k*cat (s^−1^)	*K*_m_ (mM)	*K*cat/*K*_m_ (s^−1^ mM^−1^)	Reference
Free LpAAL	13 ± 0.7	5.2 ± 0.3	2.5	[9]
Immobilized LpAAL	7.3 ± 1	6.3 ± 0.5	1.2	This study

## Data Availability

The data are included within this article.

## Abbreviations

The following abbreviations are used in this manuscript:

AAL	Aspartate ammonia-lyase
CS	Chitosan
*E. coli*	*Escherichia coli*
IPTG	Isopropyl β-D-1- thiogalactopyranoside
LpAAL	*Lactobacillus paracasei* aspartate ammonia-lyase
MBP	Maltose binding protein
PVA	Polyvinyl alcohol
SDS	Sodium dodecyl sulfate
